# Male triploid oysters of *Crassostrea gigas* exhibit defects in mitosis and meiosis during early spermatogenesis

**DOI:** 10.1002/2211-5463.13356

**Published:** 2022-06-21

**Authors:** Floriane Maillard, Nicolas Elie, Nadège Villain‐Naud, Mélanie Lepoittevin, Anne‐Sophie Martinez, Christophe Lelong

**Affiliations:** ^1^ Unité de Formation et de Recherches (UFR) des sciences Université de Caen Normandie France; ^2^ Biologie des Organismes et Ecosystèmes Aquatiques (BOREA) FRE2030 Museum National d’Histoire Naturelle (MNHN) Centre National de la Recherche Scientifique (CNRS) Institut de Recherche et Développement (IRD) Sorbonne Université (SU) Université de Caen Normandie (UCN) Université des Antilles (UA) Paris Cedex France; ^3^ Centre de Microscopie Appliquée à la Biologie SF4206 Interaction Cellule‐Organisme‐Environnement (ICORE) Université de Caen Normandie France; ^4^ Oestrogènes et Reproduction (OeReCa) EA2608 Université de Caen Normandie France

**Keywords:** *Crassostrea gigas*, meiosis, mitosis, oysters, spermatogenesis, triploid

## Abstract

The Pacific oyster, *Crassostrea gigas,* is a successive irregular hermaphrodite mollusk which has an annual breeding cycle. Oysters are naturally diploid organisms, but triploid oysters have been developed for use in shellfish aquaculture, with the aim of obtaining sterile animals with commercial value. However, studies have shown that some triploid oysters are partially able to undergo gametogenesis, with numerous proliferating cells closed to diploids (3n alpha) or a partial one with an accumulation of locked germ cells (3n beta). The aim of our study therefore was to understand the regulation of spermatogenesis in both groups of triploid oysters (alpha and beta) from the beginning of spermatogenesis, during mitosis and meiosis events. Our results demonstrate that the reduced spermatogenesis in triploids results from a deregulation of the development of the germinal lineage and the establishment of the gonadal tract led by a lower number of tubules. Morphological cellular investigation also revealed an abnormal condensation of germ cell nuclei and the presence of clear patches in the nucleoplasm of triploid cells, which were more pronounced in beta oysters. Furthermore, studies of molecular and cellular regulation showed a downregulation of mitotic spindle checkpoint in beta oysters, resulting in disturbance of chromosomal segregation, notably on spindle assembly checkpoint involved in the binding of microtubules to chromosomes. Taken together, our results suggest that the lower reproductive ability of triploid oysters may be due to cellular and molecular events such as impairment of spermatogenesis and disruptions of mitosis and meiosis, occurring early and at various stages of the gametogenetic cycle.

## Graphical abstract

The aim of this study was to understand the molecular and cellular regulations that take place at certain checkpoints of the cell cycle, in mitosis and meiosis during early spermatogenesis, in both groups of triploid oysters (alpha and beta).

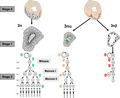

**Abbreviations**
2n: diploid
3n: triploid
4n: tetraploid
APC/C: anaphase‐promoting complex
bub3: budding uninhibited by benomyl 3
cdc20: cell division cycle 20
EF1α: elongation factor 1α
GA: gonadal area
GAI: Gonadal Area Index
GAPDH: glyceraldehyde‐3‐phosphate dehydrogenase
GT: gonadal tubules
GTI: Gonadal Tubule Index
H3S10p: H3-phosphoS10 antibody
HP1: heteroprotein 1
IHC: fluorescent immunohistochemistry
LBR: Lamin B receptor
mad2l1: mitotic arrest-deficient 2-like 1
MCC: mitotic checkpoint complex
mis12: kinetochore complex component
rad21: a component of the cohesin complex
SAC: spindle assembly checkpoint
ST: storage tissue
TAI: Tubule Area Index

The Pacific cupped oyster, *Crassostrea gigas* (Thunberg, 1793), is a successive and irregular hermaphrodite, predominantly of male gender during the first year and then able to change sex at each reproductive season [1]. Its protandry has recently been questioned [2]. Its gonad corresponds to a network of gonadal tubules imbricated in a connective tissue including the storage tissue. Its breeding cycle is annual and seasonal with four different stages of gametogenesis, which were described by Berthelin et al. [4]; Franco et al. [5]. During the sexual resting stage (stage 0), only some undifferentiated germ cells with no mitotic activity are present in the gonadal tubules, which do not allow sex identification. Stage 1 is characterized by gonial proliferation, and the sex of individuals can be determined by histological methods at the end of the stage. During stage 2, successive differentiations (active spermatogenesis or oogenesis) conduct the gonadal cells to stage 3, corresponding to the mature reproductive stage [4, 5, 6].

Triploid (3n) oysters were first introduced in the 1980s [7] for purposes of shellfish aquaculture. Now, triploid oysters have become an important part of aquaculture, thanks to their ability to grow faster than diploid (2n) animals, their supposed lack of gametogenesis [8, 9, 10], and their superior taste quality [11, 12]. Their great performance in terms of growth is due to the reallocation of energy to the growth of somatic tissues at the expense of the growth and differentiation of the gonad [8, 9, 10, 13]. Nevertheless, it has been proven that many triploid oysters are not totally sterile even if their reproductive capacities are significantly reduced compared to diploid animals [14, 15] Among them, 3n alpha shows an active gametogenesis‐like diploid animals but low gametes at sexual maturity stage while 3n beta showed a locked gametogenesis with an accumulation of abnormal gonia at stage 1 and only few mature gametes at sexual maturity [14]. The sterility triploid oysters are therefore partial and very heterogeneous depending on the animals. However, this heterogeneity is poorly documented in triploid oysters as is their gametogenesis. Triploid gametogenesis is insubstantially documented as well as its reproduction. This heterogeneity may be due to environmental factors such as temperature, photoperiod, and food availability [15, 16, 17]. Another explanation for this diversity could come from the different methods of inducing triploidy and from the selection of the genitors [1, 18].

The first method used to induce the triploids consists of blocking the expulsion of polar bodies with the cytochalasin B or with other cell cycle inhibitors [19]. The animals obtained as a result of this technique of induction are called ‘chemical’ triploid oysters [20, 21]. This chemical method has a number of disadvantages including its incomplete effectiveness and the significant toxicity of the chemical used. It was therefore abandoned to be replaced by a more natural method, that is, the cross‐breeding between tetraploid and diploid animals. The resulting offspring are named ‘natural’ triploid oysters [11, 19]. For this purpose, tetraploid (4n) oysters are produced by chemical induction techniques by inhibiting the expulsion of polar body I in oocytes of triploid fertilized oysters and then fertilizing these oocytes with haploid sperm. Although this method triggers the production of viable 4n oysters that reach sexual maturity [9, 22], in the end it only produces 80–90% triploid embryos, when it should theoretically produce 100% [9, 11]. In order to increase the induction rate, a new method was developed. It consist in producing tetraploid oysters by inhibiting the expulsion of polar body II with cytochalasin B in diploid oocytes and then fertilizing these oocytes with diploid sperm from tetraploid males [10, 23]. This method is still currently used to produce triploid oysters [10].

However, the induction of polyploidy can cause many changes at the molecular, chromosomal, and cellular levels, resulting in phenotypic variations [24]. Indeed, the presence of an additional set of chromosomes in triploid animals may be critical during biological processes such as mitosis and meiosis that undergo cell divisions [25]. It may then impact the levels of transcripts, especially during the regulatory mechanisms of gene dosage compensation [26, 27]. During the mitosis of polyploid cells, impaired mitotic spindle assembly can cause a chaotic segregation of chromatids and the production of aneuploid cells [27, 28]. The transcriptomic profiling of gametogenesis has shown that such disruptions of the mitosis can be responsible for the impaired gametogenesis observed in triploid oyster [29]. All triploids displayed a downregulation of genes associated with cell division, and the comparison of 3n alpha and 3n beta transcriptomes with 2n revealed the involvement of a cell cycle checkpoint during mitosis in the successful but delayed development of gonads in 3n alpha oysters [29].

The aim of this study is to understand the molecular and cellular regulations that take place at certain checkpoints of the cell cycle, in mitosis and meiosis during gametogenesis, in both groups of triploid oysters (α and β), focusing only on male oysters. This study also aims to reach a finer understanding of the differences in the structure of the gonad which are at the origin of their differences in terms of reproductive effort. In order to do this, we compared α and β triploid oysters with diploid ones. First, we assessed the reproductive effort by means of a histological quantitative approach focusing on the number and area of gonadic tubules. We also highlighted molecular expression profiles by means of a transcriptomic quantification method in order to explore the regulation of the spermatogenesis. Finally, the dynamic and remodeling of the chromatin was assessed during mitosis and meiosis occurring during the gametogenetic cycle.

## Materials and methods

### Animals and rearing conditions

Diploid and triploid juveniles (*Crassostrea gigas*) in their 1st year of life were provided by a commercial farm (Calvados, France). Triploid oysters were produced by crossing tetraploid males with diploid females. Oysters were maintained on field at Cricqueville en Bessin (Calvados) between December 2017 and May 2018. The animals were sampled every 15 days until May, and after that monthly until September 2018. For each individual, pieces of gonad and gill tissues were sampled, frozen in liquid nitrogen, and stored at −80 °C for total RNA extraction and qPCR. A transverse section of gonad was made and fixed in Davidson’s solution for histology and immunocytochemistry.

### Ploidy determination

Ploidy was determined individually for both diploid and triploid oysters by using a statistically representative group of 25 animals for each ploidy. From each animal, a piece of gills was dissected and the ploidy was certified by flow cytometry. To do this, a Muse Cell Cycle Kit with a Guava Muse Cell Analyzer (Luminex, Austin, TX, USA) was used following the manufacturer’s instructions. The DNA content was determined by propidium iodide labeling from a cell suspension provided to centrifugate shredded gills in the final volume of cold 70% ethanol.

### Histological analysis

Transverse sections of gonad were fixed in Davidson’s solution (10% glycerol, 20% formaldehyde, 30% ethanol (95%), 30% sterile seawater, 10% acetic acid). They were then dehydrated in ethanol and embedded in paraffin wax. Three‐micrometer‐thick sections were stained with Prenant‐Gabe trichrome [30]. The gametogenetic stages and sex were determined *a posteriori*. For diploid oysters, it was based on the criteria described by Refs [4] and [5]: stage 0 (sexual rest, undifferentiated cells), stage 1 (gonia proliferation), stage 2 (maturation; spermatogenesis or oogenesis), and stage 3 (ripe gonads before spawning). For 3n oysters, α and β patterns were determined according to Ref. [14]: stage 0 (sexual rest as in 2n), stage 1 (α‐pattern: gonia proliferations as in 2n and β‐pattern with highly disturbed gametogenesis, with numerous germ cells locked in prophase of mitosis) (Appendix S1), stage 2 (α‐pattern: maturation as in 2n and β‐pattern still exhibiting locking events), stage 3 (α‐pattern: mature animals closely resembling 2n and β‐pattern nearly completely sterile, with only a few gametes) (Appendix S2).

### Quantitative analysis of reproductive effort

To evaluate the reproductive effort, each of the histological sections was scanned at 20× (0.25 µm·pixel^−1^) using a ScanScope CS microscope slide scanner (Leica Biosystems, Nanterre, France). For each image, a downsampling by 8 was applied to allow an analysis with Python algorithms [31] and using the ilastik software [32]. A color normalization [33] of all images was first carried out to enhance the performance of image analysis algorithms. Then, three different tissues were detected on the images: the whole tissue section without the gills that had previously been removed, the gonadal tubules (GT) and the storage tissue (ST). The whole tissue area was extracted, thanks to a threshold intensity component of the color space ‘Hue, Saturation and Intensity’. GT areas were obtained with a threshold on components L* and a* of color space ‘CIE L*a*b*’, where L* represents the lightness from black to white, a* represents colors ranging from green to red, and b*represents the colors ranging from blue to yellow. ST areas were obtained with a pixel classification method using the ilastik software [32]. The areas of these three different tissues were computed in pixels so as to access the reproductive effort of diploid and triploid oysters, using gonadal area (GA) measurement. This area, located around the digestive gland, contains GT and ST and is known to vary in proportions depending on the gametogenetic stage, in diploid oysters as in triploid oysters. Here, to be even more precise, the Gonadal Tubule Index (GTI) and the Tubule Area Index (TAI) were specifically measured. The GTI represents the percentage of GA in relation to the whole animal cross section [GTI = (GA/surface of the whole cross section) * 100] and TAI is the percentage of GT in relation to the whole animal cross section [TAI = (GT/surface of the whole cross section) * 100]. The average number of tubules per cross section and the mean area per tubule was also measured in group of animals at the beginning of the gametogenetic stage (stages 0 and 1). Area (*A*), circularity, perimeter (*P*), major and minor axes, and compacity of each tubule were obtained. The sorting of the tubules was carried out using the Gravelius compactness coefficient: $K=P\frac{P}{2\sqrt{\pi A}}A=0.28\frac{P}{A}$ with the threshold of K < 1.7 to keep only the tubules with circular cross sections [34, 35]. To ensure that the number of these tubules was representative of the totality of the tubules, we have compared their proportion to that of all tubules for each animal and no significant difference was found.

### RNA Extraction

Ten individual gonad samples and three gill samples for gametogenetic stage of diploid and triploid oysters were used for the quantitative expression analysis. The tissues were grounded in 500 µL of TriReagent (Sigma‐Aldrich, Saint Quentin Fallavier, France). One hundred microliters of bromo‐3‐chloropropane (Sigma‐Aldrich) was then added, and the samples were vortexed before centrifugation (12 000 ***g***, 10 min, 4 °C). The aqueous phase was retrieved and the total RNA extraction was performed using a NucleoSpin RNA Clean‐up Kit (Macherey‐Nagel, Hoerdt, France), following the manufacturer’s instructions. RNA concentration, purity, and integrity were checked by spectrophotometry (NanoDrop ND‐1000, Thermo Scientific, Waltham, MA, USA). To prevent genomic DNA contamination, RNA samples were treated with DNAse I (1 U·µL^−1^ of total RNA, Sigma). Then, the total RNA was reverse‐transcribed to prepare for the cDNA. Two hundred and fifty nanograms of total RNA from each sample was reverse‐transcribed using 200 U of MMuLV‐RT (Moloney Murine Leukemia Virus Reverse transcriptase, Promega, Charbonnières les bains, France) in the presence of 12 U of RNase inhibitor (RNasin, Promega), 5 mm of RNAse free dNTPs, and 100 ng of random primers in the appropriate buffer (Promega) at 37 °C during 90 min. cDNA was stored at −20 °C until further usage.

### Quantitative real‐time PCR (qPCR)

The expression of mRNA in oysters during the gametogenetic cycle was investigated using a quantitative real‐time PCR analysis. The primer sequences were designed using the Primer 3 software (Table 1) and synthesized by Eurogentec (Seraing, Belgium). The reference gene, elongation factor 1α (EF1α), was selected among two housekeeping genes [EF1α and glyceraldehyde‐3‐phosphate dehydrogenase (GAPDH)]. The high stability of this housekeeping gene was previously identified by Refs [36, 37] and was further confirmed in the present study. The qPCR mix contained 1X *GoTaq SYBR Green Mix* (Promega), 5 ng cDNA, and 15 µm of forward and reverse primers in a final volume of 15 µL. The amplification conditions consisted of one cycle at 95 °C for 5 min, followed by 45 cycles of 95 °C for 15 s and 60 °C for 45 s. The specific amplification of the target sequence was estimated from the melting temperature curves. The relative expression level of each target gene was calculated based on the threshold value (*C*_t_) deviation of this target gene from the housekeeping gene (EF1α).

**Table 1:** List of names, sequences, and corresponding gene names and NCBI ID numbers (*: reference gene) of the primers used for quantitative real‐time PCR analysis in *Crassostrea gigas*. ‘F’ and ‘R’ indicate, respectively, forward and reverse primers.

Primer	Nucleotide sequences (5'‐3')	Gene name	NCBI Gene ID
EF1α‐F	ACGACGATCGCATTTCTCTT	Elongation factor 1‐alpha*	105338957
EF1α‐R	ACCACCCTGGTGAGATCAAG		
GAPDH‐F	TTGTCTTGCCCTCTTGC	Glyceraldehyde‐3‐phosphate dehydrogenase*	105340512
GAPDH‐R	CGCCAATCCTTGTTGCTT		
CDC20‐F	TACCAAAGGACCAGGCATGC	*Cell division cycle 20*	105332569
CDC20‐R	GTGTTTTCGCCGTTGTACTGA		
MAD2L1‐F	ACCACGTCAGAAGTCAGAGA	*Mitotic arrest‐deficient 2‐like 1*	105340350
MAD2L1‐R	CAGCAGGGGTAGGAATGTGA		
MIS12‐F	GGACAGGGTACAGATGACACA	*MIS12 Kinetochore Complex Component*	105330856
MIS12‐R	GCTATGATGTGTTTACGGAGCT		
RAD21‐F	TGCCATCAACACTTTCCTTCAG	*RAD21 Cohesin Complex Component*	105337371
RAD21‐R	ACCCGTGCAATCTTTTCCAC		
BUB3‐F	GTTGTGTCGAGTACTGCCCA	*BUB3 Budding uninhibited by benomyl 3*	105332696
BUB3‐R	CTTGTCCGGTTGAGTGAAGGA		

### Statistical analysis

The gene expression levels and the measures assessed by quantitative histology between diploid and triploid oysters at each gametogenetic stage were illustrated on graphs by means and SEM. The results were analyzed using a Student’s parametric *t*‐test with a *P*‐value < 0.01. All analyses were performed with the prism.v6 (GraphPad) software.

### Immunohistochemistry

Five‐micrometer sections were deparaffinized, rehydrated, and washed in 1X phosphate‐buffered saline (PBS) during 10 min. The sections were permeabilized with 200 µL of 0.1% Triton X‐100 in 1X PBS for 10 min at room temperature. Antigen retrieval was performed with 200 µL of freshly made 2N hydrochloric acid in 1X PBS in a 37 °C incubator during 30 min. After denaturation, nonspecific sites were blocked with an incubation in 100 µL of 0.1 m Tris/HCl (pH8.3) for 10 min followed by one in 500 µL of blocking solution [0.25% bovine serum albumin (BSA), 0.5% Triton X‐100 in 1X PBS] for 1 h at room temperature in a humid chamber. After that, the slides were incubated overnight with a rabbit polyclonal phospho‐histone H3 (Ser10) antibody (Millipore 06–570, Millipore Corporation, Guyancourt, France) diluted (1 : 500 previously validated by Cavelier et al. [38]) in a blocking solution at 4 °C in a humid chamber. The next day, the sections were washed three times (15 min each) with 0.1% PBS Tween‐20 and then incubated during 1 h with a secondary antibody, Donkey Anti‐Rabbit IgG H&L (1 : 1000) (Alexa Fluor 594) (ab150080) and with Hoechst 20 mm (1 : 1000) in a blocking solution at room temperature. Three further washes with 0.1% PBS Tween‐20 were then performed, and the slides were mounted in mounting medium (Ibidi, Sigma‐Aldrich). The image analysis was performed with a FV1000 confocal laser scanning biological microscope (Olympus, Rungis, France).

## Results

### Reproductive effort during spermatogenesis

**Fig. 1:**
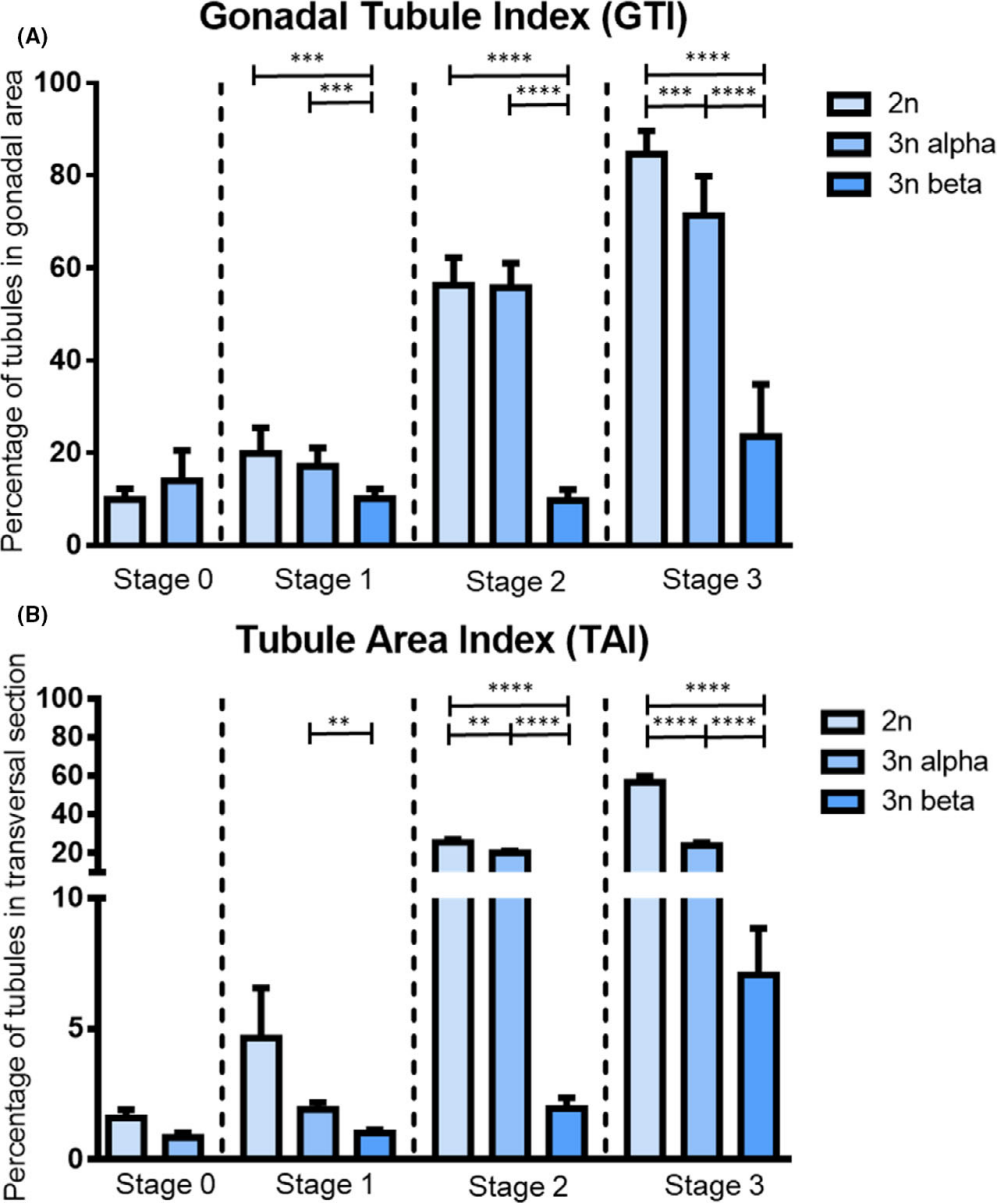
Quantification of reproductive effort in male *Crassostrea gigas* oysters. Assessed through the Gonadal Tubule Index(GTI) (A) and the Tubule Area Index (TAI) (B) in a transverse sections of diploid (*n* = 36) and triploid (*n* = 64) oysters during the gametogenic cycle (stage 0: resting period, stage 1: gonia proliferations, stage 2: maturation, and stage 3: mature animals). By means of a statistical analysis (Student's test) comparing triploid and diploid animals during the gametogenetic cycle, the significant differences are indicated by asterisks (****P < 0.0001, ***P < 0.0005 and **P < 0.001). Bars represent standard error of mean.

**Fig. 2:**
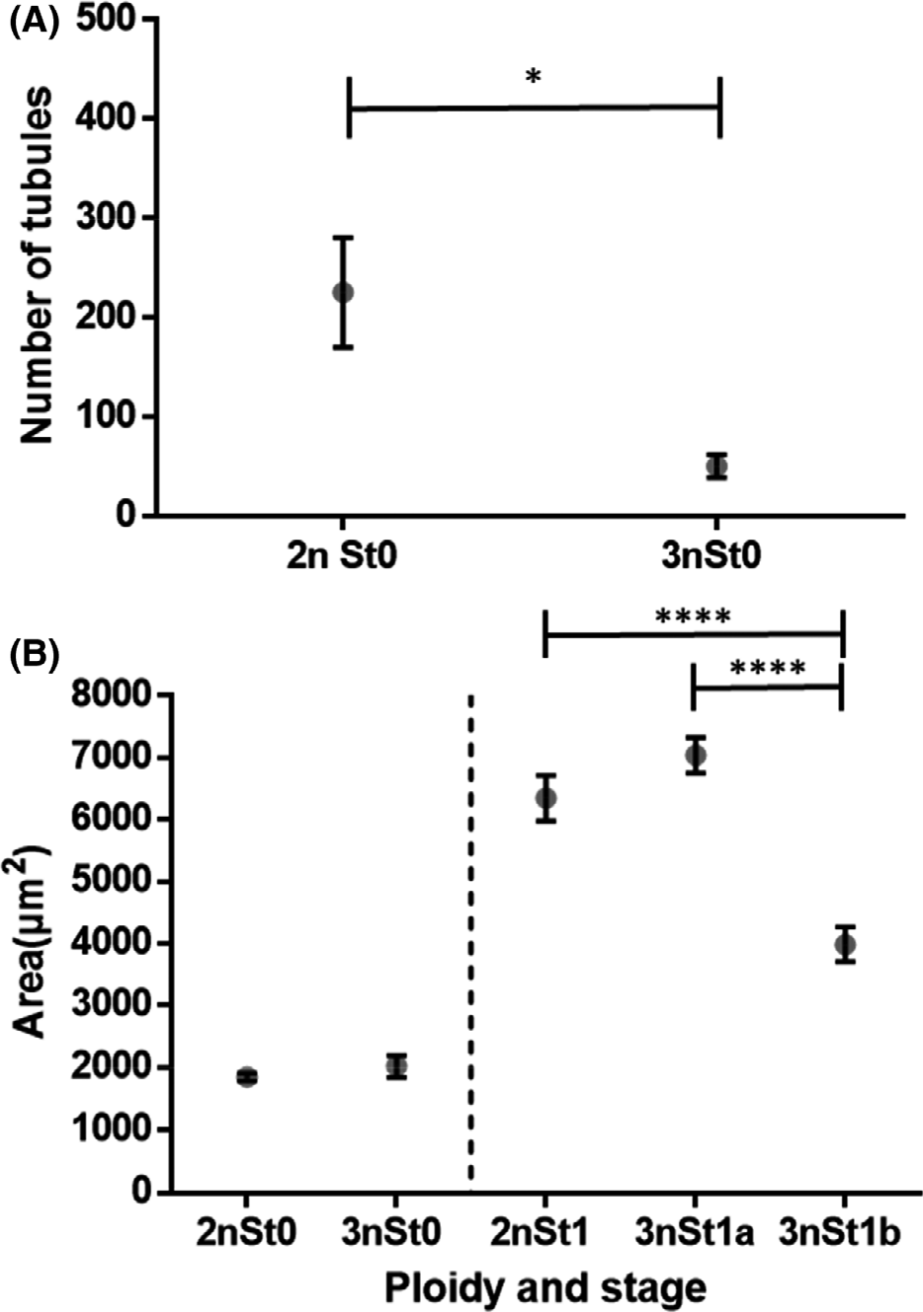
Quantification of the average number of gonadal tubules (A) and the mean area per tubule (B) in a transverse section of male diploid (2n) (*n* = 12) and triploid (3n) (*n* = 27) oysters during the gametogenic cycle (stage 0: resting period, stage 1: gonia proliferations). By means of a statistical analysis (Student's test) comparing triploid and diploid animals at each gametogenetic cycle, the significant differences are indicated by asterisks (****P < 0.0001 and *P < 0.05). Bars represent standard error of mean.

The reproductive effort during the gametogenetic cycle was studied by quantitative histology analysis on transversal sections of male diploid and triploid oysters. It was first measured as the percentage of gonadal area occupying the whole cross section (GTI) (Fig. 1A). During the quiescent stage (stage 0), while alpha and beta triploids cannot be distinguished, no significant difference was observed between 2n and 3n animals, with around 10–15% of the gonadal area. At stage 1, GTI was 20% for diploid, 17% for alpha triploid, and 10% for beta triploid oysters. At the end of the gametogenetic cycle (stage 3), it reached 85% in diploid, 71% in alpha triploid, and 23% in beta males triploid. A pairwise comparison within each gametogenic stage found significant differences between diploid and alpha and beta triploid oysters from stage 1 to stage 3. Whatever the stage, the GTI of 3n beta was always significantly lower in comparison with diploid and alpha triploid males. The GTI of alpha triploids was also significantly lower compared with diploid oyster, but only at stage 3. A fine analysis was also made to quantify more precisely the occupation of the tubules in the whole animal section through the Tubule Area Index (TAI) (Fig. 1B). In comparison with diploid oysters, the TAI was significantly reduced in triploid animals at stages 2 and 3. At stage 3, it was significantly reduced by half in alpha triploid oysters and by eight in beta triploid male oysters. The TAI not only takes into account the number of tubules but also their surface, which increases during gametogenesis, according to the filling rate of the tubules by germ and somatic cells. Thus, a complementary analysis focusing on the number of tubules and on the mean area per tubule was performed in order to find out whether the decrease in TAI in triploids could be due to a decrease in the number of tubules and/or in their surface. This analysis was only conducted at stages 0 and 1, when the tubules are clearly individualized on the section (Fig. 2). These stages are also relevant because (a) stage 0 corresponds to the very beginning of the gametogenesis, illustrated by scarce tubules filled with very few cells, and (b) stage 1 of gonia proliferation was described as a crucial stage to understand gametogenesis in triploids [14]. This analysis showed that at stage 0, diploid animals have a significantly higher number of tubules per section than triploid males (Fig. 2A), although the mean tubule area was similar for both animals (Fig. 2B). At stage 1, diploid and alpha triploid oysters presented the same mean values of tubules areas, while beta triploid oysters highlighted a significantly reduced mean area per tubule compared with both diploid and alpha triploid oysters (Fig. 2B), in line with the results of the GTI (Fig. 1A).

### Molecular regulations during spermatogenesis

**Fig. 3:**
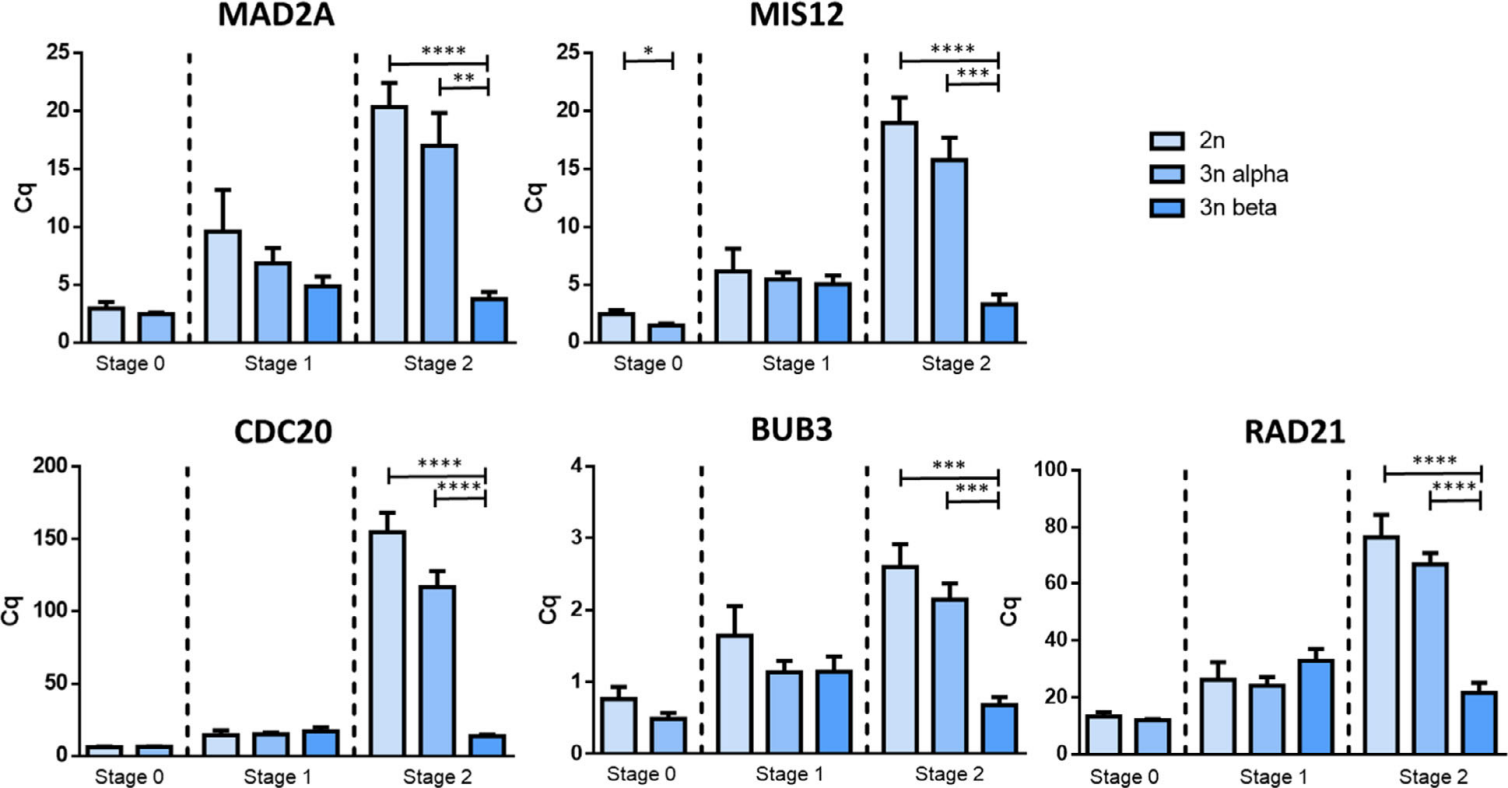
Quantitative PCR analysis of relative mRNA expressions of MAD2L1, MIS12, CDC20, BUB3, and RAD21 in gonad of male diploid and triploid *Crassostrea gigas* during the gametogenetic cycle (stage 0: resting period, stage 1: gonia proliferations, stage 2: maturation). Each bar represents the mean of expression level of the target transcript replicates (*n* = 10) related to the housekeeping gene EF1α. By means of a statistical analysis (Student's test) comparing triploid and diploid animals at each gametogenetic cycle, the significant differences are indicated by asterisks (****P < 0.0001, ***P < 0.0005, **P < 0.001 and *P < 0.05). Bars represent standard error of mean.

Here, the objective was to gain more insights into the regulatory gene network involved in the gametogenetic disruption in alpha and beta triploid oysters. For this purpose, genes were selected among those which appeared dysregulated between diploid and triploid oysters in a previous study carried out by Refs [29, 36] and using large‐scale approaches. In order to gain insight into the genes involved in gametogenetic disruption beta oysters, several ones have been selected from the previous studies from Refs [29, 36], appearing dysregulated between the 2n and 3n animals. We especially focused on those involved in the kinetochore structure and its regulation during mitotic and meiotic progression, namely mad2l1 (mitotic arrest‐deficient 2‐like 1), mis12 (kinetochore complex component), cdc20 (cell division cycle 20), bub3 (budding uninhibited by benomyl 3), and rad21 (a component of the cohesin complex). This meant performing an expression analysis by quantitative PCR on diploid and triploid male gonads at stage 0 of sexual rest and at stages 1 and 2 when mitosis and meiosis take place, respectively (Fig. 3). In diploid and alpha triploid oysters, the trend for all transcripts corresponded to a progressive increase through the gametogenesis, from stage 0 to stage 2. In contrast, in beta triploid oysters, despite an increase in all gene expressions between stages 0 and 1, a significantly lower expression was highlighted at stage 2 compared with diploid and alpha triploid oysters. Such significantly lower mRNA expression in triploids compared with diploids was also observed for MIS12 in stage 0.

### Cell divisions and chromatin remodeling along the cell cycle during early spermatogenesis

**Fig. 4:**
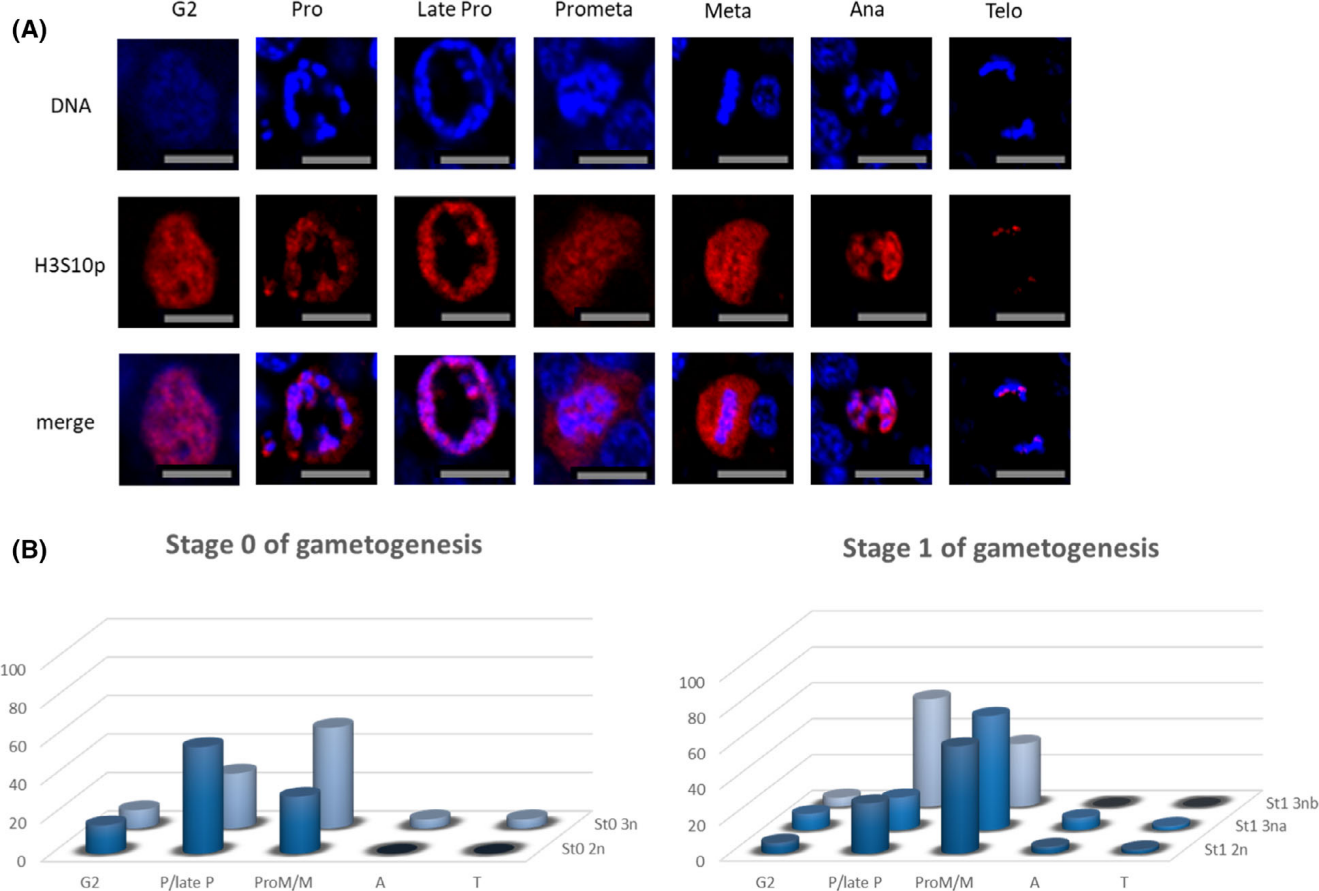
Immunofluorescence labeling of H3S10p (red) at different stages of mitosis in cross sections of gonadal tubules of male diploid oysters during stage 1 of the gametogenetic cycle. DNA was stained with Hoechst (blue) (A). Relative frequency of each figure of mitosis during the early stages of gametogenesis (stages 0 and 1) of diploid and triploid oysters (B). Scale bar: 5 µm.

**Fig. 5:**
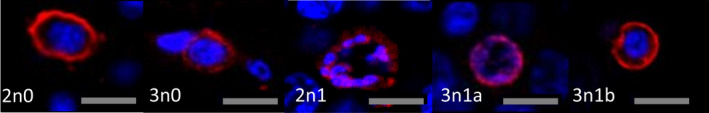
Germ cells at prophase in male diploid (2n) and triploid (3na: 3n alpha and 3nb: beta) oysters during early stages of gametogenetic cycle (0: stage 0 and 1: stage 1). DNA was stained with Hoechst (blue). Scale bar: 5 µm.

**Fig. 6:**
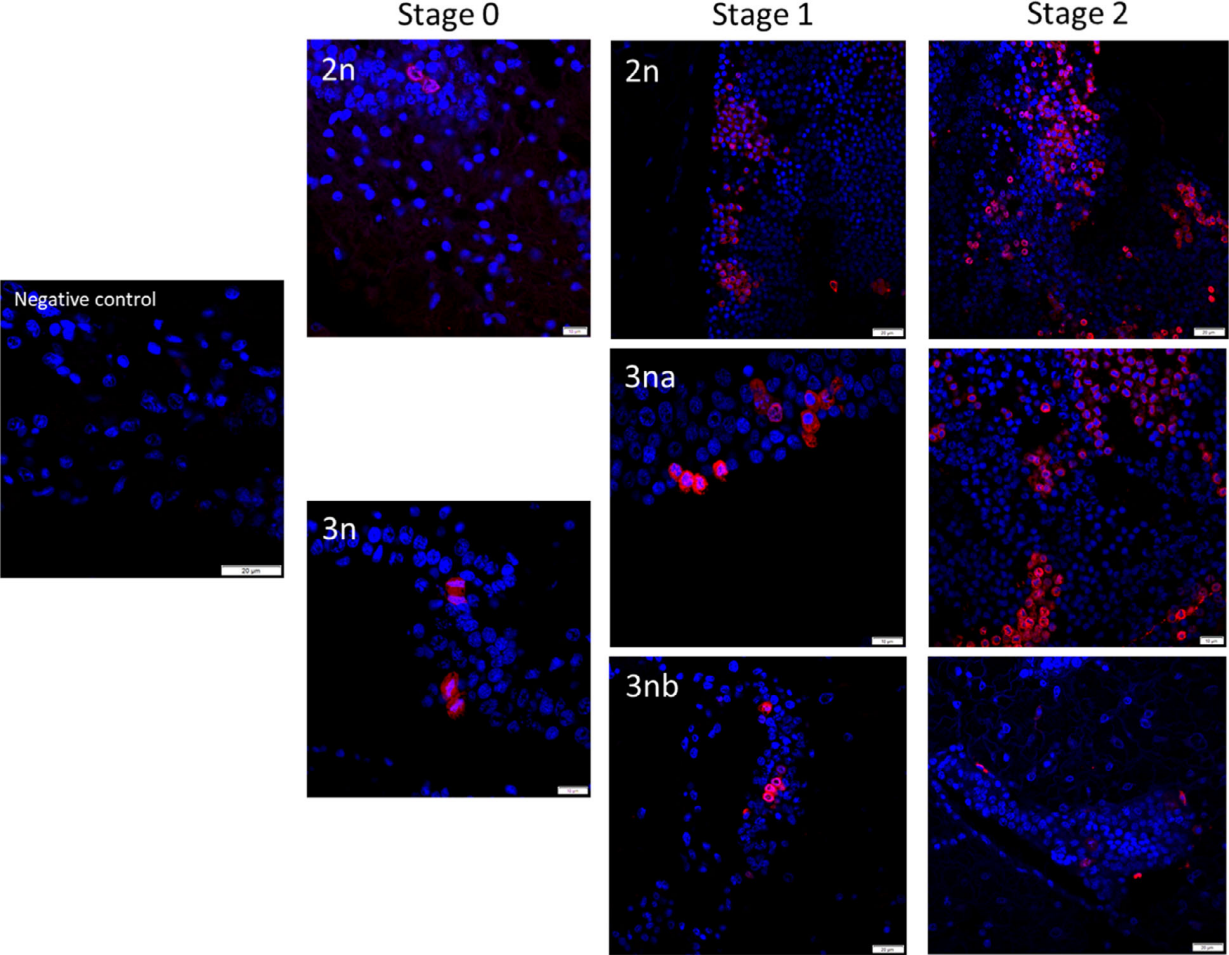
Immunofluorescence labeling of H3S10p (red) in cross sections of gonadal tubules in male diploid (2n) and triploid (3na: 3n alpha and 3nb: beta) oysters during the gametogenetic cycle (stage 0: resting period, stage 1: gonia proliferations and stage 2: maturation). DNA was stained with Hoechst (blue). Scale bar: 20 µm.

To follow the cell divisions at the origin of male gonial proliferations in diploid and triploid oysters, we traced the dynamic distribution of the chromatin at stage 1, when mitoses are most frequent, but also just before at stage 0 and then at stage 2 when meiosis occurs. For that purpose, the histone H3 phosphorylated on the Serine 10 residue was detected by fluorescent immunohistochemistry (IHC), using a H3‐phosphoS10 antibody (H3S10p) in association with a DNA staining with Hoechst dye. H3S10p‐specific signal was certified by the lack or low labeling in Hoechst‐stained nuclei in the storage tissue (tissue with low or reduced mitotic activity) and on the negative controls using no primary antibody. Confocal images revealed an intense signal associated with the stained chromatin in the gonadal tubules, with special and temporal variations depending on the mitotic stages (Fig. 4). Thus, a staining was observed starting from the G2 phase until the telophase (Fig. 4A). At the G2 phase, the Hoeschst staining (blue) and the H3S10p staining (red) were diffuse and they overlapped each other through the whole nucleus. Then, they progressively reached the periphery of the nucleus to finally become perinuclear in the late prophase. At the prometaphase, when the chromosomes start to condense and at the metaphase when all chromosomes compact on the equatorial plate, the H3S10p signal overlapped with condensed chromosomes but this pattern could also be observed outside the chromosome localization area. At the anaphase, when sister chromosomes are getting apart after centromeric fission, both signals were overlapping again. At the telophase, when the separated chromosomes started to uncoil and to become less condensed, the immunofluorescence appeared as aligned patches, localized on the chromosomal areas. When the relative frequency of each mitosis stage was counted at stages 0 and 1 and compared between diploid and triploid oysters (Fig. 4B), it appeared that at stage 0 of gametogenesis, most of the cells in diploid and triploid oysters were in the late prophase (63% for diploids and 30% for triploids) and in the prometaphase/metaphase (30% for diploids and 55% for triploids). However, diploids were rather in the late prophase whereas triploids were in the prometaphase/metaphase. Nonetheless, at this stage, the number of mitosis figures is low. In stage 1 when cells proliferate, when alpha and beta triploids can be distinguished, the cells in mitosis were still predominantly in the late prophase and prometaphase/metaphase. Interestingly, diploids and alpha triploids were rather in the metaphase whereas beta triploid oysters were in the late prophase. Looking specifically at the signal distribution in prophase (Fig. 5), at stage 0, the same staining was observed in diploids and in triploids, with heterochromatin localized at the center of the nucleus while H3S10p staining was observed at its periphery. In contrast, at stage 1, the staining was different in beta triploid animals compared with diploid and alpha triploid oysters. In these last two cases, heterochromatin was observed at the periphery of the nucleus and was overlapping with the H3S10p staining while in beta triploid heterochromatin was localized at the center of the nucleus while H3S10p staining was observed at its periphery. When looking more broadly within the tubules and later on during the gametogenetic cycle (Fig. 6), it appeared that the cells labeled H3S10p were (a) less numerous in triploid oysters (especially beta triploid) than in diploid ones whatever the gametogenetic stage and (b) still present at stage 2, which encounters meiosis.

## Discussion

### Could disturbances in the establishment of the gonadal tubules and in the gametogenesis during stages 0 and 1 of spermatogenesis explain the lower reproductive effort of beta triploid oysters*?*

Two strategies of gametogenesis were previously described, in triploid oysters by Jouaux et al. [14], a α‐pattern, corresponding to animals displaying numerous proliferating gonia at stage 1, resulting in abundant gametes at stage 3 and a β‐pattern, which was associated with a locked gametogenesis and only few mature gametes at sexual maturity. The authors supported these results by demonstrating, at stage 3, a lower reproductive effort in triploid oysters compared to diploid, using the Gonadal Area Index (GAi) and the Gonadal Tubule Index (GTi). In the present work that specifically focuses on triploid males, the same both patterns were observed (results not shown). In contrast, in Jouaux et al. [14], the reproductive effort was assessed from stage 0, at various stages of the gametogenetic cycle, and not only with GAi and GTi but also by estimating the average number of tubules per cross section and the mean area per tubule. Thus, in this study, at stage 3 of sexual maturity, as stated by Jouaux et al. [14], the GTI and TAI were lower in alpha triploids and beta triploids compared with diploid oysters, thus confirming the idea of a lower reproductive effort at this stage of sexual maturity. In alpha triploids, such differences were explained by Guo and Allen [9] and Gong et al. [18] by differential fecundities between diploid sand triploids, although they made a distinction between alpha and beta. Jouaux et al. [14] disagreed, since they observed equivalent fecundities between diploids and alpha triploids. In beta triploids, our results also demonstrated that the lower reproductive effort occurred very early, as soon as stages 0 or 1. According to our results, this could be explained by a lower number of tubules as observed at stage 0 in triploid oysters compared with diploid, but also at stage 1 by a significantly lower mean area per tubule (knowing that the number of tubules is not lower; results not shown). At this stage, and knowing that tubules are filled with proliferating gonia, a lower mean area per tubule may suggest an impaired gametogenesis with the presence of fewer germ cells within the tubules. Indeed, this latter idea is supported by previous work done by Jouaux et al. [14] who mentioned the presence of numerous germ cells locked in the mitosis prophase at stage 1 in beta triploid oysters, thus suggesting a stop in the gametogenesis at this point. Taken all together, our results suggest that the lower reproductive effort observed in beta triploid oysters may be due to disturbances occurring as soon as stage 0 or 1 of the reproductive cycle. These disturbances would occur during the establishment of the gonadal tubules and during germ cells mitosis occurring during spermatogenesis.

### Could chromatin remodeling and dysregulations of genes involved in epigenetic modifications impair mitosis in stage 1 of spermatogenesis in 3n β oysters?

In order to deepen the understanding of the cellular impairments of mitosis occurring in beta triploid oysters early during spermatogenesis, we performed an immunofluorescence labeling of the phosphorylated histone H3 on the serine 10, using a H3S10p antibody. This marker is associated with each step of mitosis and meiosis. The expression pattern of H3 is exposed to dynamic changes during spermatogenesis [39]. In our study, the frequencies of each mitosis phase measured at stages 0 and 1 of the reproductive cycle showed that beta triploid males were locked between the end of the prophase and the start of the prometaphase. These results are in line with those observed by Jouaux et al. [14]; that is, the gonia is locked in the prophase in beta triploid oysters. Previous studies have demonstrated a link between the histone H3 phosphorylation and chromosome segregation during the cell division process. The histone H3 is phosphorylated at Ser 10 during the prophase, with a peak level at the metaphase, and it is dephosphorylated at the anaphase to get out for of the division cycle [40, 41]. A mutation of this phosphorylation in the ciliated protozoan *Tetrahymena thermophile* causes condensation and segregation defects, suggesting that the H3 phosphorylation is correlated with chromosome condensation during mitosis and meiosis [42]. Moreover, underphosphorylation of histone H3 has also been connected to aberrant chromosome behavior during mitotic and meiotic division in insects [43]. In our study, in diploid oysters, during mitosis, chromatin staining was usually colocalized with H3S10p staining, except at the prometaphase and metaphase, when chromatin was highly condensed. These results also highlight a link between the histone H3 phosphorylation and chromatin remodeling during mitosis. Besides, this remodeling could be explained, as suggested by some authors, [44] by an increase in negative charge due to the phosphorylated histone H3 that makes it dissociate from chromosomal DNA, therefore causing chromatin condensation. In the same way, in beta triploid male oysters, the particular pattern of germ cells locked between the late prophase and the beginning of the metaphase could be explained by a dysregulation of the histone H3 phosphorylation followed by an abnormal condensation of the germ cell nuclei. This hypothesis is also supported by our proteomic results (not shown) which highlighted a significant downregulation of the expression level of protein histone H3 in beta triploid oysters and suggests a dysregulation at the post‐translational level. Further studies examining post‐translational modifications of histones are therefore necessary in order to maintain this hypothesis. Our results of H3S10p staining at stage 2 of the gametogenesis (meiotic stage) also suggest that the blocking of the germ cells persists throughout the gametogenetic cycle, in contrast with the results of Ref. [8] who only suggested a delay. Our results also showed a particular pattern of chromatin localization in prophase in beta triploid males only, at the center of the nucleus while the H3S10p staining was localized at its periphery. Indeed, the frequent organization of the nucleus has two types of heterochromatin: constitutive and facultative at the periphery of the nucleoplasm, near or associated with lamina and euchromatin at central position. A unique exception to chromatin organization in eukaryotes is found in rod photoreceptor cells of nocturnal mammals [45]. In these cells as in the germ cells of beta triploid males, the positions of eu‐ and heterochromatin are inverted in the nuclei. This peculiar chromatin remodeling happens during the development and the cellular differentiation. In beta triploid male oysters, this peculiar chromatin localization during mitosis could be associated with the peripheral labeling of H3S10p. Indeed, in rod cells it is associated with major post‐translational modifications of the histones, mainly the epigenetic marks H3K9me3 and H4K20me3 and with nuclear tether proteins, Lamin‐A/C and LBR (Lamin B Receptor) [46, 47]. These latter two proteins are involved in the tethering of LAD domains in association with HP1 (heteroprotein 1) and H3K9me2/3. LBR is used to attach the nuclear lamina to the inner nuclear membrane and to bind HP1‐associated heterochromatin. It acts in association with H4K20me2PRR14, which requires Lamin‐A/C for localization at the inner nuclear periphery [48]. The loss of tethering of the nuclear lamina induces alterations of the genome architecture [49]. Surprisingly, in 2n oysters, a similar central localization of chromatin and peripheral localization of the Histone H3p were also observed in germ cells at stage 0 (resting gametogenetic stage). This startling localization requires further studies. However, in mice, a role of epigenetic modifications during very early spermatogenesis was recently mentioned, when prospermatogonia differentiate into differentiating or undifferentiated spermatogonia after birth [50]. A demethylation of H3K9me2 by the JMJD1A and B demethylases would be at the origin of this differentiation of spermatogonia [50]. Taken all together, our results therefore suggest (a) that epigenetic modifications appear essential to the progression of spermatogenesis in diploid oysters and (b) that their probable deregulation might be involved in the blocking observed in beta triploid oysters.

### Could dysregulations in genes involved in the spindle assembly checkpoint (or SAC) be a sign of impaired meiosis at stage 2 of spermatogenesis in 3n β oysters?

The frequent infertility of triploid individuals is a general pattern directly related to their ploidy, as mentioned for numerous species. It is frequently due to irregular chromosomal pairing and perturbed segregation during mitotic and meiotic events [20, 51, 52, 53] In our study, the mRNA expression of genes establishing the normal spindle‐kinetochore interaction during cell divisions was assessed by qPCR. A significantly lower expression of these genes was observed at stage 2 in beta triploid male oysters, suggesting their downregulation during mitosis and meiosis.

**Fig. 7:**
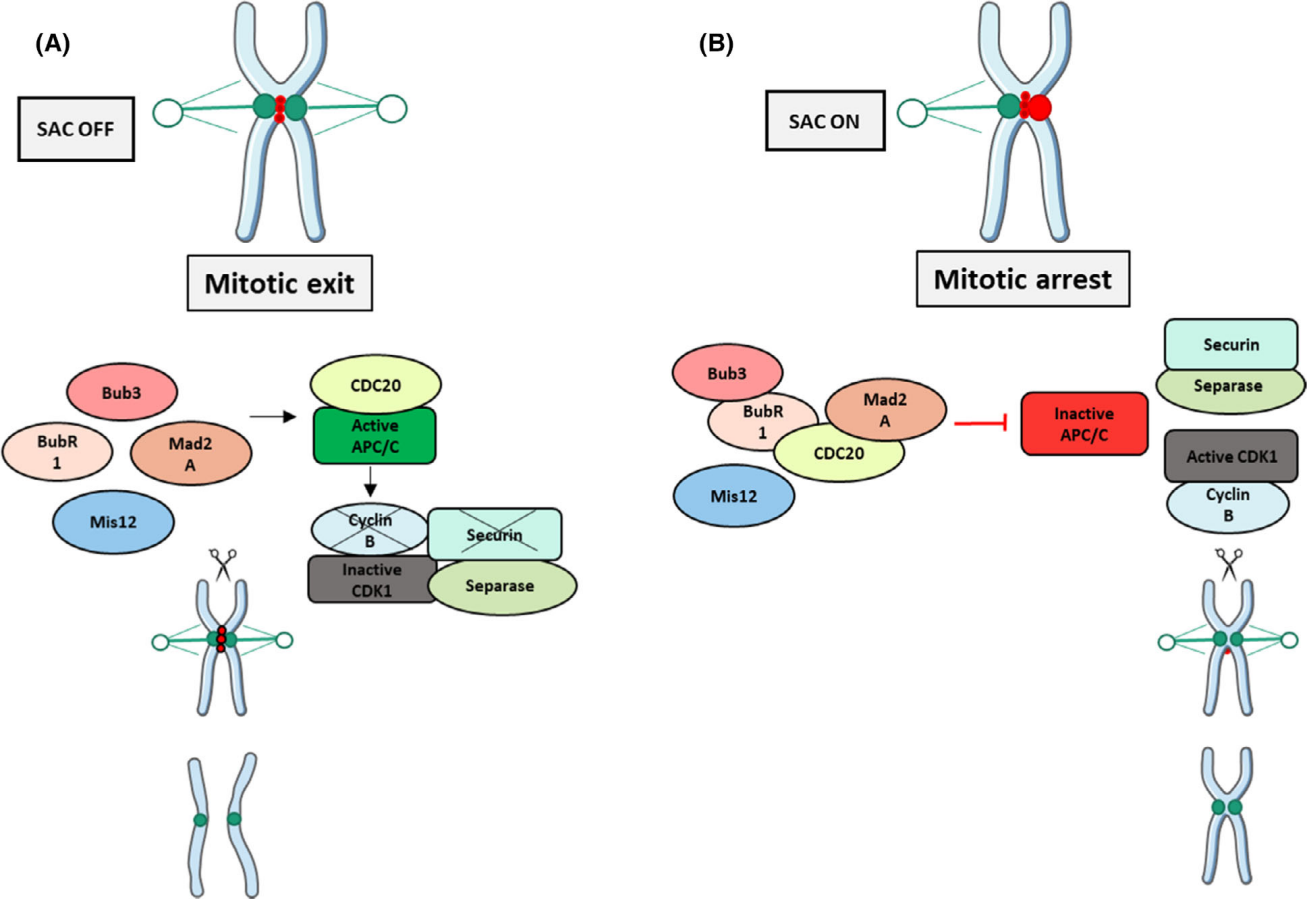
Speculative model for the coordination of the metaphase–anaphase transition. Mitotic and meiotic chromosome segregation is controlled by spindle assembly checkpoint (SAC). (A) The unpaired and mismatched kinetochores activate the SAC signals and block the cell cycle by keeping the anaphase‐promoting complex (APC/C) inactive. (B) A correct and stable microtubule’s attachment is sufficient for SAC silencing and mitotic exit via the activation of the APC/C.

The segregation of the chromosomes during the mitosis or meiosis requires their attachment to the kinetochore, a large protein complex which ensures the assembly of the centromere of each chromosome to the spindle of microtubules. This complex is controlled by a molecular surveillance mechanism that checks the correct microtubule attachment, named spindle assembly checkpoint (or SAC) (Fig. 7). During equational meiosis (as during mitosis), and once all centromeres are associated with the microtubules, the SAC is silenced, therefore allowing the mitotic exit via the activation of the APC/C (Fig. 7A). More precisely, when all kinetochores are correctly associated with the microtubules at the prometaphase, the activation of APC/C^CDC20^ promotes Cyclin B and Securin proteolysis by the proteasome. Thus, by inhibiting the APC/C, the MCC stabilizes these substrates and their destruction induces a mitotic exit [54, 55]. Then, the separation of sister chromatids takes place under the control of a cohesion protease separase such as Rad21 [56, 57]. During the unpaired attachment of the microtubules to the kinetochore, the SAC signal is activated and locks the cell cycle by inactivating the anaphase‐promoting complex (APC/C) (Fig. 7B). Then, the SAC acts through numerous effectors forming the mitotic checkpoint complex (MCC). From their effectors of the MCC, the three SAC proteins Mad2l1, BubR1, and Bub3, in interaction with CDC20, participate to the formation of the outer kinetochores, that is, protein complexes formed by numerous proteins including Mis12. They bind and then inhibit the APC/C^CDC20^ complex required to prevent the entry into the anaphase. Based on our results, we can posit the hypothesis of a potential impairment during meiosis in beta triploid male oysters. During the first division, homologous chromosomes may be held together through chiasmata that are supported by cohesion along chromosome arms. Sister kinetochores would be associated with each other and thus co‐oriented. Homologous kinetochores would be pulled by spindle microtubule into the opposite pole (homologous biorientation). The homologous recombination of chromosomes during meiosis would make the kinetochore’s attachment to the chromosome more difficult [58]. In male oysters, this step may occur at stage 2 of the gametogenesis, when germ cells differentiate from gonia to spermatozoa. In beta triploid oysters, the downregulation of SAC actors at stage 2 could reduce the chances of spindle chromosome attachment. In addition, the decrease in cdc20 expression level would lead to a decreased activation of APC. Maintaining SAC activation could result in a cell cycle arrest and lock the entry of the cell into the anaphase during the meiosis. Therefore, a deregulation of the meiotic checkpoint may induce an impairment in the chromatids segregation in beta triploid oysters. Furthermore, a downregulation of the cohesion rad21 may induce chromosome instability, thus amplifying the locking of germ cell in beta triploid oysters. All this would therefore prevent the germ cells from completing their meiosis. In contrast, in alpha triploid oysters, the expression profile of the kinetochore actors was similar to that of diploid oysters, which would suggest an activation of the APC, and therefore, a normal segregation and a meiosis exit.

Then, our work allowed us to assume that the lower reproductive effort of beta triploid male oysters may be (a) first due to disturbances in the establishment of the gonadal tubules and to a locking of germ cells due to a misregulation of the chromatin remodeling by modifications of epigenetic marks, during quiescent and mitotic stages of spermatogenesis (stages 0 and 1) and (b) in a second time, to an aberrant segregation of chromosomes during the meiosis due to the perturbation of the SAC mechanism. Hence, these results lead to a more detailed study of the actors of the chromatin structure, through the analysis of the post‐translational modifications of histones (methylation, acetylation essentially) and associated proteins (as isoforms of HP1, LBR, Lamins) and their expression during the complete spermatogenesis. It is also important to follow the proteomic expression of the kinetochore actors.

## Conflict of interest

The authors declare no conflict of interest.

## Author contributions

CL and FM designed the experiment. FM, NE, NVN, ML, and CL performed the experiments. FM, ASM, and CL analyzed the data. FM, CL, and ASM wrote and edited the manuscript, and ASM and CL supervised the project.

## Data accessibility

The data presented in this study will be made available upon reasonable request.

## Supporting information


**Appendix S1.** Histological cross sections of *Crassostrea gigas* male gonadal area at stage 1 of gametogenesis (gonial proliferation). The gonadal area is composed of the gonadal tubule (GT) and of the storage tissue (ST). Diploid (A) and alpha triploid male oysters (B) present a proliferation of germinal lineage in the gonadal tubule with the figures of mitosis (M) especially for diploid oyster whereas beta triploid oyster (C) exhibit locking events (clear cytoplasmic area: asterisk and condensed nuclei: arrow).

**Appendix S2.** Gametogenetic stages in male diploid (2n) and triploid (3nα: 3n alpha and 3nβ: beta) oysters. (stage 0: sexual resting period, stage 1: gonial proliferation, stage 2: maturation, and stage 3: sexual maturity).
