# Supplementary figures and images for: Male triploid oysters of Crassostrea gigas exhibit defects in mitosis and meiosis during early spermatogenesis
Source: FEBS Open Bio. 2022 Jun 21;12(8):1438–52. doi: 10.1002/2211-5463.13356 (PMC9340784; doi:10.1002/2211-5463.13356)

*
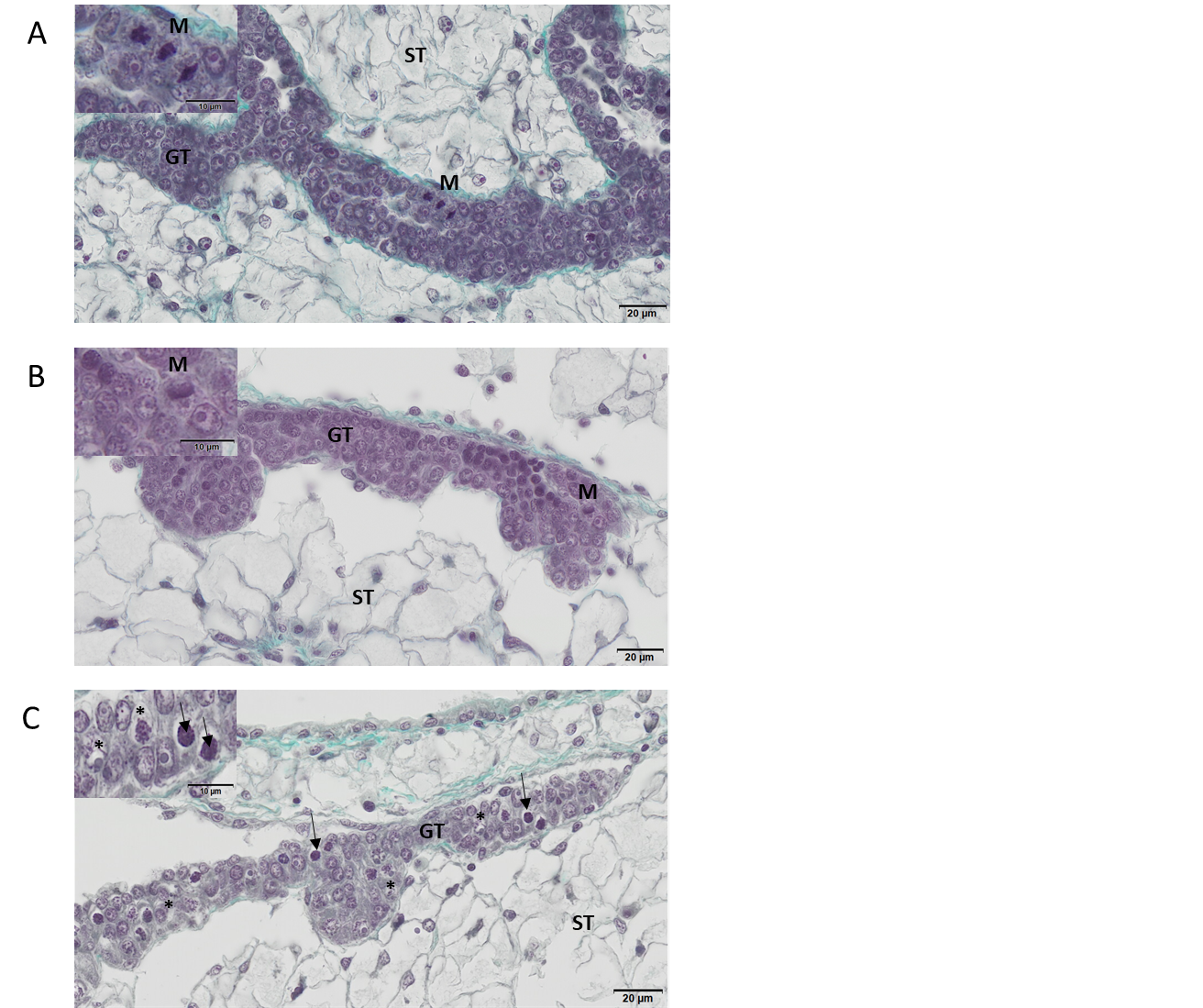
*

Supplement: Supplementary file 1 — Appendix S1. Histological cross sections of Crassostrea gigas male gonadal area at stage 1 of gametogenesis (gonial proliferation). The gonadal area is composed of the gonadal tubule (GT) and of the storage tissue (ST). Diploid (A) and alpha triploid male oysters (B) present a proliferation of germinal lineage in the gonadal tubule with the figures of mitosis (M) especially for diploid oyster whereas beta triploid oyster (C) exhibit locking events (clear cytoplasmic area: asterisk and condensed nuclei: arrow). [file FEB4-12-1438-s002.docx]

**
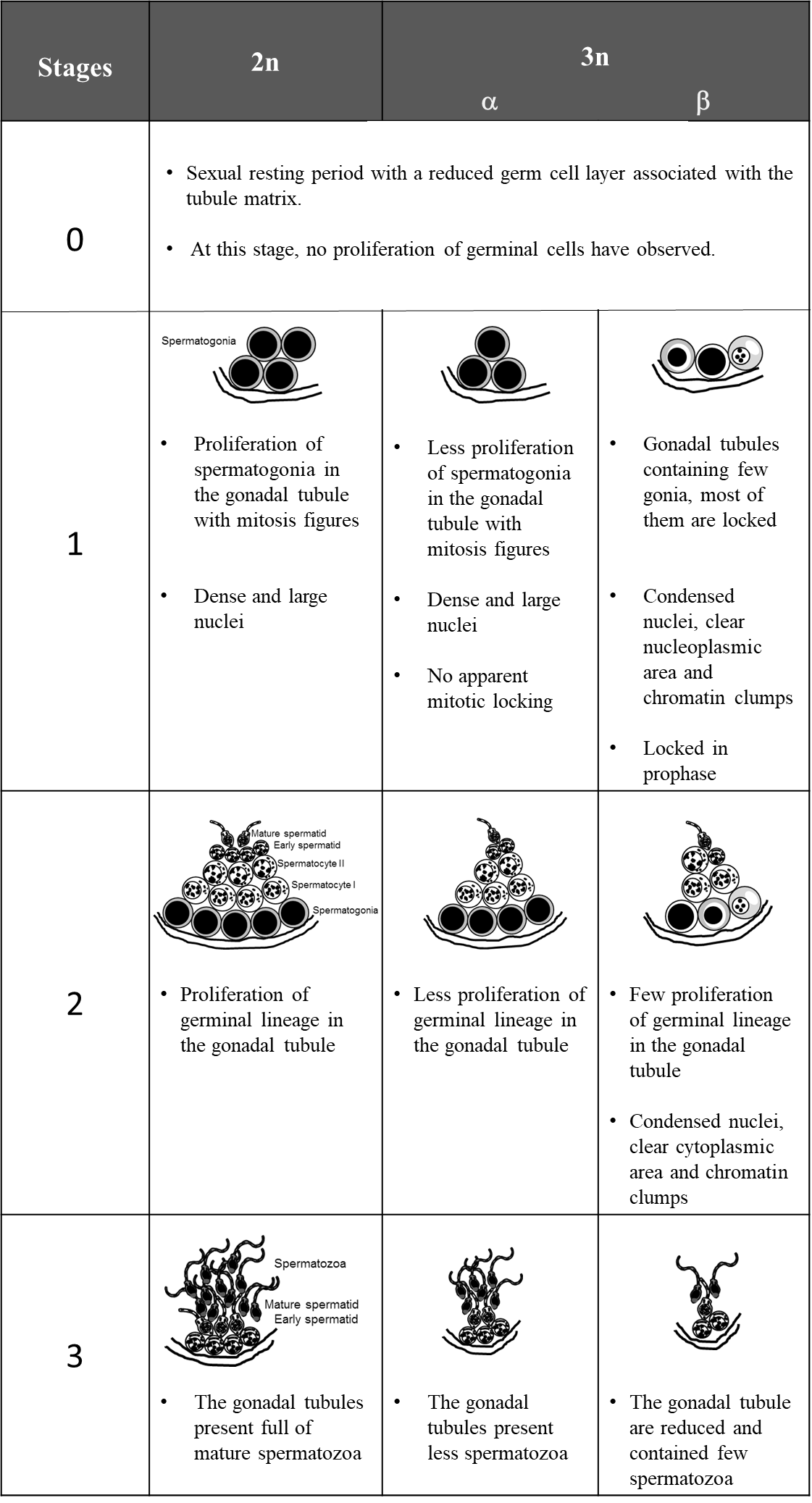
**

Supplement: Supplementary file 2 — Appendix S2. Gametogenetic stages in male diploid (2n) and triploid (3nα: 3n alpha and 3nβ: beta) oysters. (stage 0: sexual resting period, stage 1: gonial proliferation, stage 2: maturation, and stage 3: sexual maturity). [file FEB4-12-1438-s001.docx]
